# COVID-19-Induced Myopathy and Diaphragmatic Weakness: A Case Report

**DOI:** 10.7759/cureus.38515

**Published:** 2023-05-03

**Authors:** Patrik Schmidt, Tobechukwu Okobi, Irhoboudu D Atogwe, Gabriel Alonso, Edwin Pena, Misbahuddin Khaja

**Affiliations:** 1 Internal Medicine, Icahn School of Medicine at Mount Sinai/BronxCare Health System, New York City, USA; 2 Internal Medicine/Pulmonary Critical Care, Icahn School of Medicine at Mount Sinai/BronxCare Health System, New York City, USA

**Keywords:** viral myopathy, covid myopathy, critical illness polyneuropathy, acute illness myopathy, covid induced ards, autoimmune myopathy, sars-cov-2, diaphragmatic weakness, critical illness myopathy, covid-19

## Abstract

Coronavirus disease 2019 (COVID-19) is a respiratory illness caused by the severe acute respiratory syndrome coronavirus 2 (SARS-CoV-2) virus that can induce myopathy, which can evolve into potentially life-threatening muscle weakness, including diaphragmatic paralysis. We present a case report of a 57-year-old female treated in the medical ICU for acute respiratory distress syndrome (ARDS) triggered by active COVID-19 infection, who subsequently developed worsening respiratory weakness from underlying COVID-19 myopathy manifesting as respiratory muscle weakness. Our patient’s muscle biopsy highlights the development of muscle atrophy without evidence of inflammatory myopathy, making the presence of pre-existing autoimmune myopathy unlikely. While literature cites different biochemical etiologies for the development of myopathy, the exact mechanism behind this phenomenon is not yet defined.

## Introduction

Coronavirus disease 2019 (COVID-19) is a respiratory illness caused by the severe acute respiratory syndrome coronavirus 2 (SARS-CoV-2) virus that has been shown to induce myopathy, which can evolve into potentially life-threatening muscle weakness, including diaphragmatic paralysis [1-6]. While literature cites different biochemical etiologies for the development of myopathy, the exact mechanism behind this phenomenon is not yet defined. Our case explores the presentation of COVID-19-induced myopathy leading to respiratory muscle weakness without evidence of any underlying inflammatory muscle disease on muscle biopsy but with evidence of muscle fiber atrophy. We also discuss different types of myopathies and the most common etiologies.

## Case presentation

Our patient was a 57-year-old female with a past medical history of hypertension and obesity, who came to the emergency room with complaints of shortness of breath and chest tightness during the high COVID-19 pandemic. She described having these symptoms for the past week, with a gradual worsening in dyspnea and generalized muscle weakness leading to her visit. She denied chest pain, recent immobilization, surgery, travel, contraception use, or any history of cancers, deep vein thrombosis, or previous pulmonary emboli. She had no smoking history or any pertinent family history.

Upon examination in the emergency room, she was hypoxic, with a resting room air saturation of 90% with moderate work of breathing, which subsequently improved to 95% after placing her on 2 liters of the nasal cannula. She was otherwise normotensive and afebrile. The patient's initial neurological exam revealed no cranial nerve deficits; however, she had generalized weakness in both upper and lower extremities, with strength graded at 3/5 in the upper extremities and 4/5 in the lower extremities. Her sensation was intact in all limbs. Her labs were significant for acute kidney injury, denoted by an elevated creatinine of 3.2 (baseline 0.9), along with high inflammatory markers low-density lipoprotein at 486 (standard 100-190 units/L), C-reactive protein at 93 (standard <5mg/L), D-dimer at 295 ng/mL (standard 0-230 ng/mL), and ferritin at 770 (standard 13-150 ng/mL). Initial chest x-ray revealed interstitial opacifications in the perihilar and lower lobe regions. The patient was admitted to the general medical unit as a person under investigation for COVID-19 and for the management of hypoxia in the setting of suspected community-acquired pneumonia. Her COVID-19 polymerase chain reaction tests came back positive.

In the ward, she was started on empiric antibiotic therapy and hydroxychloroquine therapy of 400 mg daily. She continued to experience worsening hypoxia in the following days with respiratory distress and tachypnea, leading to a need for mechanical respiratory support. She received tocilizumab and was managed for acute respiratory distress syndrome (ARDS). She was successfully extubated to non-invasive positive pressure ventilation (NIPPV) after 48 hours of mechanical ventilation but showed signs of respiratory muscle weakness, with incentive spirometry showing reduced lung volumes below 500mL suggestive of diaphragmatic weakness. Her hospital course was complicated by severe pulmonary edema and the development of acute heart failure, suspected to be secondary to COVID-19 myocarditis. Despite intravenous diuretics and preload and afterload reduction, there was no improvement in the patient’s fraction of inspired oxygen (FiO2) requirements or radiologic studies. She received convalescent plasma and completed an entire course of the antibiotic regimen. However, she continued to exhibit respiratory muscle weakness and rapid tiring off NIPPV, with subsequent development of hypoxia and tachypnea. Repeat chest x-rays showed interval worsening of bilateral pulmonary infiltrates with reduced lung volumes despite diuresis and completion of the antibiotic regimen. Given these findings, the patient was re-intubated.

Blood gas analysis at the time showed an elevated carbon dioxide level of 93 mmHg (standard 35-45 mmHg) and pH of 7.15 (normal 7.35-7.45). Her oxygen requirements have also increased dramatically to 80% oxygen content on mechanical ventilation, with a congruent decline in hemodynamic stability, leading to the necessity of pressors and fluid resuscitation in the shock setting. The patient’s persistent hypoxia, elevated work of breathing, and declining respiratory status in the ICU were believed to be associated with her progressive diaphragmatic weakness, which led the ICU team to send myositis marker work-up along with a muscle biopsy to evaluate for possible underlying autoimmune-myositis. The patent’s laboratory studies showed negative anti-mitochondrial antibodies, anti-Jo-1 antibodies, smooth muscle antibodies, and DNA antibodies. Creatine kinase levels peaked at 330 units/liter (standard 20-200 units/liter), and no severe persistent electrolyte abnormalities were noted on her chemistry panels. Muscle biopsies were taken from the patient’s left quadriceps muscle. While being managed for her ARDS, our patient suffered a cardiac arrest which led to her passing despite resuscitative efforts.

**Figure 1:**
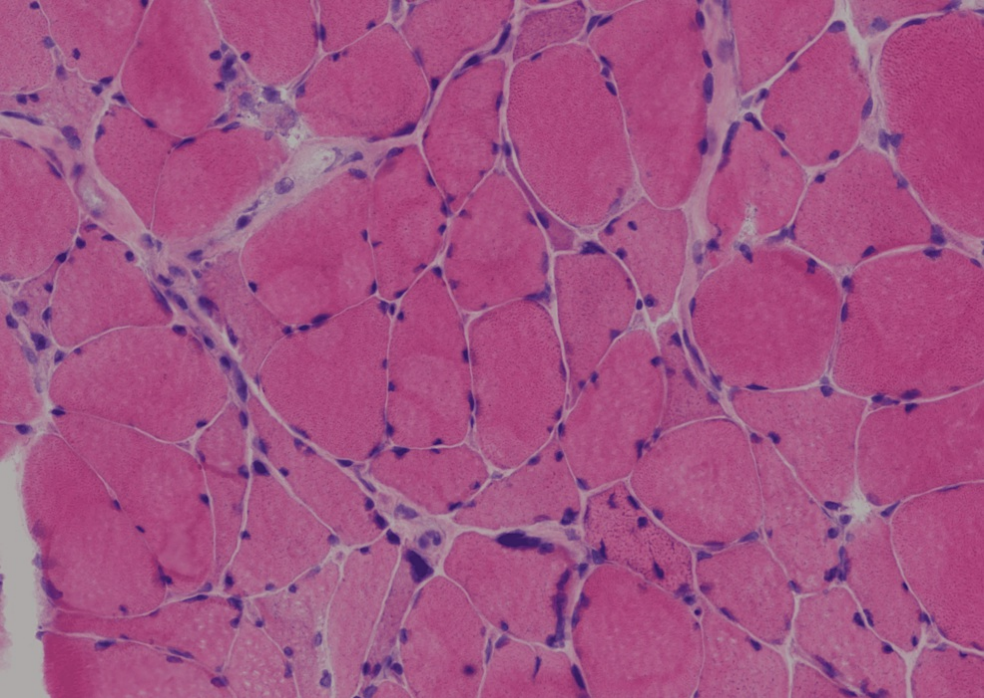
Biopsy of representative frozen sections of the skeletal muscle biopsy stained with standard hematoxylin and eosin stain and with an enzyme histochemical stain.

**Figure 2:**
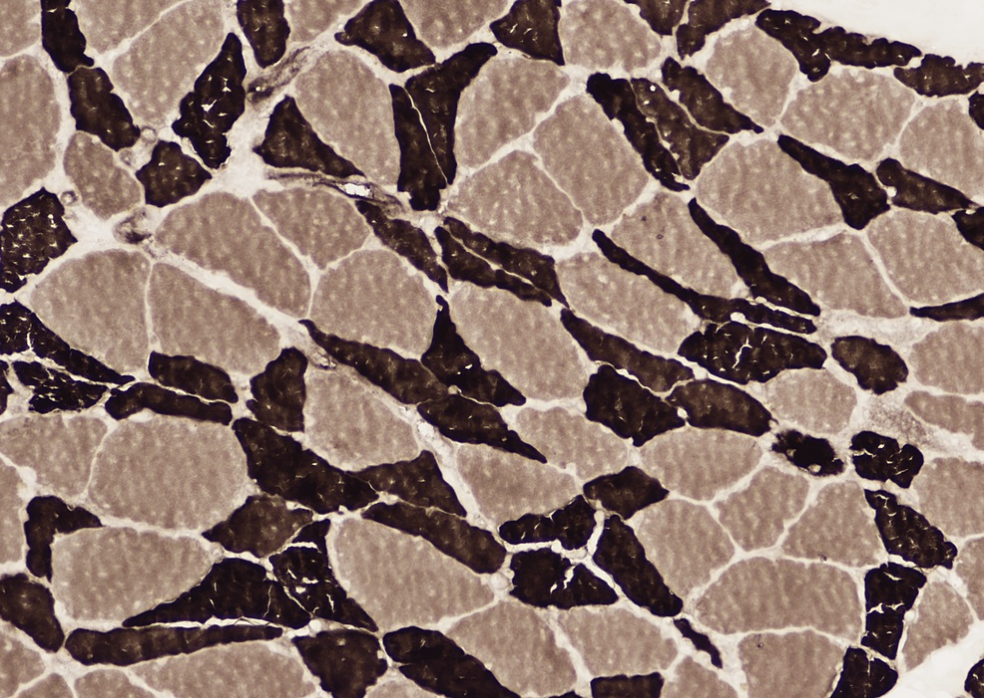
Biopsy of representative frozen sections of the skeletal muscle biopsy stained with an enzyme histochemical stain for myosin ATPase activity performed at pH 9.5. The ATPase stain highlights the type 1 myofibers as lightly stained cells while the type 2 myofibers are darkly stained.

Figures 1, 2 show representative frozen sections of the skeletal muscle biopsy stained with standard hematoxylin and eosin (H&E) stain and with an enzyme histochemical stain for myosin ATPase activity performed at pH 9.5. The ATPase stain highlights the type 1 myofibers as light-stained cells while the type 2 myofibers are darkly stained. In this patient’s biopsy, the small atrophic myofibers are predominantly darkly stained showing type 2 myofiber atrophy. The H&E stain does not show evidence of inflammatory myopathy.

## Discussion

COVID-19-induced myopathy is just one form of a broader spectrum of inflammatory muscle diseases. Other etiologies, such as idiopathic inflammatory myopathy and those that stem from endocrine disturbances and toxic substances, can also present with sudden onset progressive muscle weakness. In the case of inflammatory myopathies, a triggered immunological reaction results in muscular weakening and inflammation [7]. Certain drugs, alcohol, and environmental toxins can cause toxic myopathies [8-13], while hormonal disorders that involve high cortisol levels or low thyroid hormone levels can cause endocrine myopathies [14]. Specifically, in critical care, high levels of oxidative stress and increased muscle apoptosis, along with ischemia and axonal degeneration, can lead to the development of critical illness myopathy (CIM) and critical illness polyneuropathy (CIP).

Viral myositis occurs exclusively in the setting of viral infections, with common etiologies being enteroviruses, cytomegalovirus (CMV), Epstein-Barr virus (EBV), and human immunodeficiency virus (HIV) [15]. Typical symptoms of enterovirus-associated myopathy include symmetric, proximal muscle weakness that is more noticeable in the lower than the upper limbs [16]. Individuals with HIV-associated myopathy may also experience muscle discomfort and atrophy in addition to gradual, symmetrical muscle weakening in the proximal lower extremities and, less frequently, the upper extremities [17]. Patients with CMV-associated myopathy, however, may exhibit pain that worsens quickly over several days and more abrupt, severe muscle weakening [18]. Individuals with EBV-associated myopathy may also show abnormalities in electromyography, increased creatine kinase levels, and acute muscle discomfort and fatigue [19].

COVID-19-associated myopathy is present in up to 50% of hospitalized patients with the virus [20-21]. It is likely caused by multiple pathogenic mechanisms, including direct muscle invasion by SARS-CoV-2, systemic inflammation, cytokine release, autoimmune-mediated myositis triggered by COVID-19 infection, and corticosteroid use [8-12,22-24]. In addition, COVID-19 has been reported to cause inflammatory cardiac myopathy and, in rare cases, has been observed to cause Guillain-Barre Syndrome. Other factors, such as pre-existing hypertension, diabetes, and severe infection, were also linked with an increased risk of developing myopathy [1,2].

Our patient’s muscle biopsy highlights the development of muscle atrophy without evidence of inflammatory myopathy, making the presence of pre-existing autoimmune myopathy unlikely. Furthermore, the presence of musculoskeletal weakness even before admission to the hospital and prolonged ICU course make CIM and CIP the less likely primary pathology. CIM presents, however, with the loss of thick filaments [25], while CIP shows neurogenic atrophy with extensive type 1 and type 2 myofiber atrophy [26]. These findings do not correlate with our patient’s muscle tissue biopsy, making CIM and CIP the sole cause of our patient’s progressive respiratory muscle weakness less likely. However, their superimposed presence could have played a role in exacerbating existing muscular dysfunction.

## Conclusions

COVID-19-induced myopathy is a rare but significant complication that should be considered in patients presenting with muscle weakness or wasting, especially those with a history of COVID-19 infection. With its long list of differential diagnoses in infected patients, diagnosing without direct muscle biopsy visualization can be challenging. There are no specific therapies targeting COVID-19-induced myopathy at this time, yet due to its high prevalence, further research on this topic can help physicians better understand its pathogenesis. This may lead us closer to developing effective treatment to prevent potential long-term neuromuscular sequala of the disease.

## References

[1] Farr E, Wolfe AR, Deshmukh S (2021). Diaphragm dysfunction in severe COVID-19 as determined by neuromuscular ultrasound. Ann Clin Transl Neurol.

[2] FitzMaurice TS, McCann C, Walshaw M, Greenwood J (2021). Unilateral diaphragm paralysis with COVID-19 infection. BMJ Case Rep.

[3] Mao L, Jin H, Wang M (2020). Neurologic manifestations of hospitalized patients with coronavirus disease 2019 in Wuhan, China. JAMA Neurol.

[4] Zhou F, Yu T, Du R (2020). Clinical course and risk factors for mortality of adult inpatients with COVID-19 in Wuhan, China: a retrospective cohort study. Lancet.

[5] Hejbøl EK, Harbo T, Agergaard J (2022). Myopathy as a cause of fatigue in long-term post-COVID-19 symptoms: evidence of skeletal muscle histopathology. Eur J Neurol.

[6] Kharouf F, Kenig A, Bohbot E, Rubin L, Peleg H, Shamriz O (2023). Increased rates of idiopathic inflammatory myopathies during the COVID-19 pandemic: a single-centre experience. Clin Exp Rheumatol.

[7] Zong M, Lundberg IE (2011). Pathogenesis, classification and treatment of inflammatory myopathies. Nat Rev Rheumatol.

[8] Manzano GS, Woods JK, Amato AA (2020). Covid-19-associated myopathy caused by type I interferonopathy. N Engl J Med.

[9] Leung TW, Wong KS, Hui AC, To KF, Lai ST, Ng WF, Ng HK (2005). Myopathic changes associated with severe acute respiratory syndrome: a postmortem case series. Arch Neurol.

[10] Li Y, Li J, Ke J (2021). Adverse outcomes associated with corticosteroid use in critical COVID-19: a retrospective multicenter cohort study. Front Med (Lausanne).

[11] Veyseh M, Koyoda S, Ayesha B (2021). COVID-19 IgG-related autoimmune inflammatory necrotizing myositis. BMJ Case Rep.

[12] Piotrowicz K, Gąsowski J, Michel JP, Veronese N (2021). Post-COVID-19 acute sarcopenia: physiopathology and management. Aging Clin Exp Res.

[13] Peters S, Kley RA (2013). Toxic and drug-induced myopathies. Neuromuscular Imaging.

[14] Ruff RL, Weissmann J (1988). Endocrine myopathies. Neurol Clin.

[15] Narayanappa G, Nandeesh BN (2021). Infective myositis. Brain Pathol.

[16] (2022). Viral and Retroviral Myositis. https://www.medlink.com/articles/viral-and-retroviral-myositis.

[17] Wulff EA, Simpson DM (1999). Neuromuscular complications of HIV-1 infection. Curr Infect Dis Rep.

[18] (2022). Neuropathies Associated with Cytomegalovirus Infection. https://www.medlink.com/articles/neuropathies-associated-with-cytomegalovirus-infection.

[19] Pestronk A, Sinha N, Alhumayyd Z, Ly C, Schmidt R, Bucelli R (2019). Immune myopathy with large histiocyte-related myofiber necrosis. Neurology.

[20] Abenza-Abildúa MJ, Ramírez-Prieto MT, Moreno-Zabaleta R (2020). Neurological complications in critical patients with COVID-19. Neurologia (Engl Ed).

[21] De Santis M, Isailovic N, Motta F (2021). Environmental triggers for connective tissue disease: the case of COVID-19 associated with dermatomyositis-specific autoantibodies. Curr Opin Rheumatol.

[22] Suh J, Mukerji SS, Collens SI, Padera RF Jr, Pinkus GS, Amato AA, Solomon IH (2021). Skeletal muscle and peripheral nerve histopathology in COVID-19. Neurology.

[23] Mohammadi S, Moosaie F, Aarabi MH (2020). Understanding the immunologic characteristics of neurologic manifestations of SARS-CoV-2 and potential immunological mechanisms. Mol Neurobiol.

[24] Ellul MA, Benjamin L, Singh B (2020). Neurological associations of COVID-19. Lancet Neurol.

[25] Lacomis D, Giuliani MJ, Van Cott A, Kramer DJ (1996). Acute myopathy of intensive care: clinical, electromyographic, and pathological aspects. Ann Neurol.

[26] Zhou C, Wu L, Ni F, Ji W, Wu J, Zhang H (2014). Critical illness polyneuropathy and myopathy: a systematic review. Neural Regen Res.

